# Separation of the Ileum and Spontaneous Occlusion of the Divided Extremities

**Published:** 1877-10

**Authors:** Thos. F. Rochester


ART. II.—*Separation of the Ileum and spontaneous Occlusion of the divided extremities.* Reported to the Buffalo Medical Association by THOS. F. ROCHESTER, M. D.

On the twentieth of August, 1877, at 7 A. M., I visited Mrs. A., thirty-eight years of age. She had been my patient for many years, and had been attended by me in several very severe attacks of constipation, depending, as was thought, upon spasmodic contraction of the colon, and yielding to morphine subcutaneously, and to assafœtida internally. Mrs. A. was tall and very thin; had borne four healthy children, and had suffered from one or more premature labors. Her general health, apart from her attacks of constipation, was good. Four years since, in one of her illnesses, *a slight inguinal hernia* was discovered. Failing to reduce it by taxis under chloroform, change of posture, applications of ice and other means, I called upon Prof. J. F. Miner, who reduced it under chloroform, by long and strong manipulation. The reduction was followed by entire relief as far as pain and constipation and vomiting were concerned, but was succeeded by a large and deep slough of the skin and subjacent tissues extending down to the peritoneum. It healed in something over two months with cure of the rupture. It was a matter of friendly controversy between Dr. M. and myself as to whether the ice or the taxis had caused the slough. Especial attention is called to this point in the history, as possibly having some relation to the remarkable pathological condition to be presently mentioned.

I had last seen Mrs. A. Oct. 12, 1876, and was now much surprised and equally hurt, to learn that she had latterly been under the treatment of homeopathic practitioners, and had been very ill for the last five weeks. I found her with a greatly distended abdomen, through the thin walls of which the convolutions of the small intestines could be distinctly felt. Her pulse was small, frequent and thready. Extremities cold and nails livid; forehead very hot; temperature 105; unconscious of my presence, but evidently in great agony, and constantly crying for chloroform; of which, her husband had administered, by inhalation, about twelve ounces through the preceding night. She had for years employed both chloroform and opium when in severe pain, but not at other times. With the symptoms above enumerated, it was but too evident that a fatal issue was near. I therefore, especially under existing circumstances, refrained from administering morphine subcutaneously, and directed a suppository of three-fourths of a grain of morphine and three-fourths of a grain of alcoholic extract of belladonna, to be used and to be repeated in two hours, if the severe pain continued. This was repeated at 11 A. M. At 3 P. M. she was quiet, but unconscious. At 9 P. M. she died. Forty hours after death, post-mortem examination was made by Drs. Diehl and W. W. Miner, in the presence of myself and several other physicians. On opening the abdominal cavity, the small intestines were found enormously distended, and injected throughout their entire length, the average diameter being three inches. The large intestine was correspondingly shrunken—being but half an inch in diameter—but apparently not thickened or diseased. The cæcum and appendix vermiformis were healthy. There was no obstruction whatever in the large intestine. Evidences of considerable peritonitis were found in the presence of lymph, adhesions and serous effusion. Attention was called by Dr. Fowler to what appeared to be a perforating ulcer of the ileum. On close examination it proved to be a slough of the outer and muscular coats, not involving the lining mucous membrane. About two feet below this point the ileum came to an abrupt end, there was a tight cul-de-sac, the intestine was completely closed, and on being distended with a blow-pipe, displayed a rounded extremity with cicatricial lines. Search being made for the continuation of the intestine, another cul-de-sac was found, separated from the first about four inches, and connected with it by mesenteric attachment. It was also distended with a blow-pipe, and corresponded with the first cul-de-sac in manner of occlusion, but was only one-fourth the size of the first in diameter. This portion of the ileum was eight inches in length and terminated naturally in the head of the colon. The specimen has been carefully prepared by Dr. Phelps, and distended, and is presented for examination. It is evident that the ileum has been *separated by a space of at least four inches*, that there is no continuity of intestinal cavity corresponding with this interval, and that each end of the separated intestine is completely closed by continuous peritoneal membrane, the rounded ends showing distinct cicatricial lines. Careful search in the abdominal cavity did not reveal any foreign body or any loose intestine. There was present, however, a muddy fluid and shreddy flocculi in abundance, and this may have been the remains of the disintegrated intestine which left the wide gap. The specimen is presented as unique and most interesting. There is plenty of room for conjecture—there is no positive evidence of the real cause of the solution of continuity. It may have proceeded from the hernia, or from morbid processes resulting from it, but that seems hardly probable, as it would have to ante-date four years. It may have been caused by a recent strangulated internal and concealed hernia, but in that event we ought to be able to find distinct traces of the strangulated intestine in the abdominal cavity. While I am inclined to think that the latter conjecture is probably correct, it is to be extremely regretted that this conclusion rests on conjecture alone.

As far as I have been able to ascertain, the patient did not have a thorough evacuation of the bowels for the five weeks preceding her death. There were discharges, per rectum, but they were all watery. For the last ten days she did not retain any nourishment —vomiting was constant and persistent. Another singular circumstance remains to be mentioned. Two weeks before her death Mrs. A. seemed better, sat up in her room, *and took a ride in a carriage* with her husband, but while out was attacked with terrific pain, was immediately brought home, and did not thereafter leave her bed.

